# Is phosphorylated tau a good biomarker of synapse pathology in Alzheimer’s disease?

**DOI:** 10.1093/braincomms/fcad142

**Published:** 2023-04-27

**Authors:** Carlos A Saura, Arnaldo Parra-Damas

**Affiliations:** Departament de Bioquímica i Biologia Molecular, Institut de Neurociències, Facultat de Medicina, Universitat Autònoma de Barcelona, Bellaterra 08193, Spain; Centro de Investigación Biomédica en Red Enfermedades Neurodegenerativas (CIBERNED), Instituto de Salud Carlos III, Madrid 28029, Spain; Departament de Bioquímica i Biologia Molecular, Institut de Neurociències, Facultat de Medicina, Universitat Autònoma de Barcelona, Bellaterra 08193, Spain; Centro de Investigación Biomédica en Red Enfermedades Neurodegenerativas (CIBERNED), Instituto de Salud Carlos III, Madrid 28029, Spain

## Abstract

This scientific commentary refers to ‘Distinct brain pathologies associated with Alzheimer’s disease biomarker-related phospho-tau 181 and phospho-tau 217 in *App* knock-in mouse models of amyloid-β amyloidosis’ by Hirota *et al.* (https://doi.org/10.1093/braincomms/fcac286) and ‘Predictive blood biomarkers and brain changes associated with age-related cognitive decline’ by Saunders *et al*. (https://doi.org/10.1093/braincomms/fcad113).

**This scientific commentary refers to ‘Distinct brain pathologies associated with Alzheimer’s disease biomarker-related phospho-tau 181 and phospho-tau 217 in *App* knock-in mouse models of amyloid-β amyloidosis’ by Hirota *et al.* (https://doi.org/10.1093/braincomms/fcac286) and ‘Predictive blood biomarkers and brain changes associated with age-related cognitive decline’ by Saunders *et al*. (https://doi.org/10.1093/braincomms/fcad113).**

Alzheimer’s disease is the most common cause of dementia and disability in the elderly affecting currently 57 million people worldwide. The number of people suffering from the disease is expected to increase in the next decades if no effective therapies based on pathological mechanisms are developed. Classical therapeutic treatments (e.g. acetylcholinesterase inhibitors) only temporally alleviate some cognitive and behavioural symptoms but without preventing disease progression, and new disease-modifying treatments targeting amyloid-β (Aβ) (e.g. immunotherapy) show still limited therapeutic benefits in clinical studies even in mild cognitive impairment (MCI) and moderate disease stages. Importantly, any new effective disease-modifying treatment should be administered as early during disease progression as possible, which requires development of accurate and sensitive biomarkers.

An impressive research mainstream in the field has lately focused on the assessment of changes of Alzheimer’s disease-related pathological molecules in brain tissue and biofluids (e.g. CSF and blood/plasma), which has opened a flourishing period of investigation on novel brain imaging technologies and biomarkers for diagnosis and prognosis of Alzheimer’s disease.^1^ A reliable molecular biomarker that predicts later cognitive decline during physiological aging or preclinical dementia stages is undoubtedly critical for accurate disease detection and timely diagnosis, which is necessary for widening the therapeutic window and improving the design of clinical trials. With this idea in mind, several recent studies have pointed at plasma levels of Aβ42/40, phosphorylated tau (p-tau), neurodegeneration [neurofilament light (NfL)] and astrocyte reactivity [glial fibrillary acidic protein (GFAP)] molecules as promising aging-dependent biomarkers that correlate with cerebral Aβ and tau pathologies and represent the core of the new recognized ATN (amyloid/tau/neurodegeneration)-based diagnosis.^2^ Accurate analysis of these biomarkers, alone or in combination, in CSF or plasma in parallel with brain positron emission tomography (PET) of amyloid and/or tau distinguishes people at preclinical, MCI and/or dementia stages, and predicts, in some cases, future cognitive decline in cognitively unimpaired elderly individuals.^3^ Plasma p-tau at Thr 181 and 217 is positively associated with cerebral measurements of Aβ- and tau-PET imaging in Alzheimer’s disease compared to cognitively unimpaired controls or frontotemporal lobar degeneration,^4,5^ and correlate with cognitive deterioration in preclinical and symptomatic stages of the disease.^4,6^ However, despite the usefulness of p-tau as potential biomarker of Alzheimer’s disease, their predictive value for age-related cognitive decline in the absence of dementia remains largely unclear. In this regard, the recent article in *Brain Communications* by Saunders *et al*.^7^ revealed a significant association of elevated baseline plasma p-tau181, NfL and GFAP, but not Aβ42/40, with greater cognitive decline ∼10 years later, whereas increase of p-tau 181 over time predicted cognitive decline even after correcting for *APOE* status and an Alzheimer’s disease polygenic risk score. Considering the significant association of plasma p-tau 181 and 217 with cognitive decline in cognitively unimpaired individuals and the conversion to pre-symptomatic Alzheimer’s disease,^3,6–8^ p-tau species may be considered good predictors of memory loss at Alzheimer’s disease preclinical stages. Interestingly, pathological tau oligomers and mature neurofibrillary tangles is phosphorylated in multiple Ser/Thr residues, so it is reasonable to speculate that brain-derived plasma tau proteins may contain several phosphorylated sites. At present, however, the specific plasma tau species containing unique or combination of phosphorylation sites (181, 217 or others pathological residues) that represent the best predictors of cognitive decline in aging and Alzheimer’s disease are unknown. Phospho-proteomics approaches may be useful to identify specific p-tau protein species present in plasma during biological and pathological aging. This could be an important step for fine-tuning development of tau biomarkers for accurate diagnosis and prognosis in the future.

Tau phosphorylation and aggregation are dynamic processes, and its release into the CSF and blood are mediated, among others, by neuronal activity, exocytosis and secretory mechanisms, with glial cells playing an unclear role. It is interesting that p-tau 181 is accumulated in some neurofibrillary tangles, neuronal dystrophic neurites around plaques and synapses in temporal cortex of Alzheimer’s disease patients compared to aged controls.^7^ We and others demonstrated that the presence of neuronal p-tau at synapses and its accumulation in glial cells was associated with synapse dysfunction in memory neural circuits in Alzheimer’s disease brain and mouse models.^9^ However, the contribution of synaptic p-tau 181—alone or combined with other pathological-related phosphorylation sites (e.g. p-tau 217, 231, …)—to synapse pathology and memory loss is still unclear. To untangle this complex puzzle, Hirota *et al.*^10^ performed extensive immunofluorescence staining and 3D reconstructions of brain cortical sections of *App* knock-in mice. According to their results, p-tau 181, 217, 231, 202/205/208 species around amyloid plaques do not accumulate in dystrophic neurites or processes of glial cells (microglia, astrocytes and oligodendrocytes). On the contrary, p-tau 217 and 231 and 202/205/208, and to a lesser extend p-tau 181, colocalized with the postsynaptic glutamatergic marker PSD95 but not with the cytomatrix presynaptic protein Bassoon or other specific presynaptic neurotransmitter proteins of glutamatergic, GABAergic, cholinergic or serotoninergic synapses.^10^ The authors suggest that these pathological p-tau species accumulate abnormally at postsynaptic sites in response to Aβ plaques in *App* knock-in mice. In contrast, Saunders *et al*.^7^ used high resolution array tomography to detect increased accumulation of p-tau 181 at synaptophysin-labelled presynaptic terminals in the human temporal cortex from Alzheimer’s disease patients. Interestingly, they also identified p-tau 181 in astrocytes and dystrophic neurites around plaques in the temporal cortex, detecting a significant increase of plaques surrounded by astrocytes and neurites containing p-tau 181 in Alzheimer’s disease cases compared to healthy aging controls.^7^ Despite discrepancy about p-tau presynaptic localization, both studies highlight the possibility of synaptic p-tau species as potential biomarkers, adding important building blocks on our understanding of the relationship between tau and synapse dysfunction. However, they have some limitations, including the necessity to validate these findings in human brain in early vulnerable regions (hippocampus, entorhinal cortex, …) at different disease stages.

Intriguingly, the exact link between synaptic p-tau and tau in biofluids remains unclear. Is synaptic p-tau the source of tau in cerebral or peripheral biofluids? What is the role of astrocytes and microglia in synaptic tau release and accumulation in biofluids? Are similar synaptic mechanisms involved in extracellular Aβ42 release in brain and biofluids? Neuronal and synaptic activities increase tau release from neurons, promoting tau propagation across neural circuits, a result that strongly suggests that synapses can be the source or responsible, at least in part, of extracellular tau and p-tau. Extracellular tau could be captured by glial cells, such as microglia and astrocytes, that actively participate in tripartite or quadripartite synapses. Notably, p-tau 181 is elevated in astrocytes of aged people with lifetime cognitive resilience compared with those with cognitive decline.^7^ The source of p-tau in astrocytes is unknown, but it is plausible that secreted neuronal tau is captured by astrocytes, as observed in mice.^9^ Although premature, it is tempting to speculate that enhanced release of tau (and Aβ) by dysfunctional synapses and/or deficient endocytosis and clearance of tau by astrocytes and microglia could lead to extracellular tau accumulation and propagation resulting in its release to central and peripheral fluids in Alzheimer’s disease. If this is the case, synapses could be considered the source of p-tau and Aβ species used currently as fluid biomarkers for Alzheimer’s disease. This hypothetical model of neuronal toxic tau and Aβ release to circulating biofluids is depicted in Fig. 1. Remarkably, if current tau immunotherapies target synaptic tau, they may be highly beneficial for lowering trans-synaptic tau propagation and circulating tau levels. In conclusion, these recent studies provide convincing evidence that p-tau biomarkers correlate with synaptic tau accumulation and cognitive decline in both Alzheimer’s disease and non-demented cohorts, further suggesting that targeting synaptic tau pathology could be a relevant therapeutic strategy for preventing cognitive decline in Alzheimer’s disease and aging.

**Figure 1:**
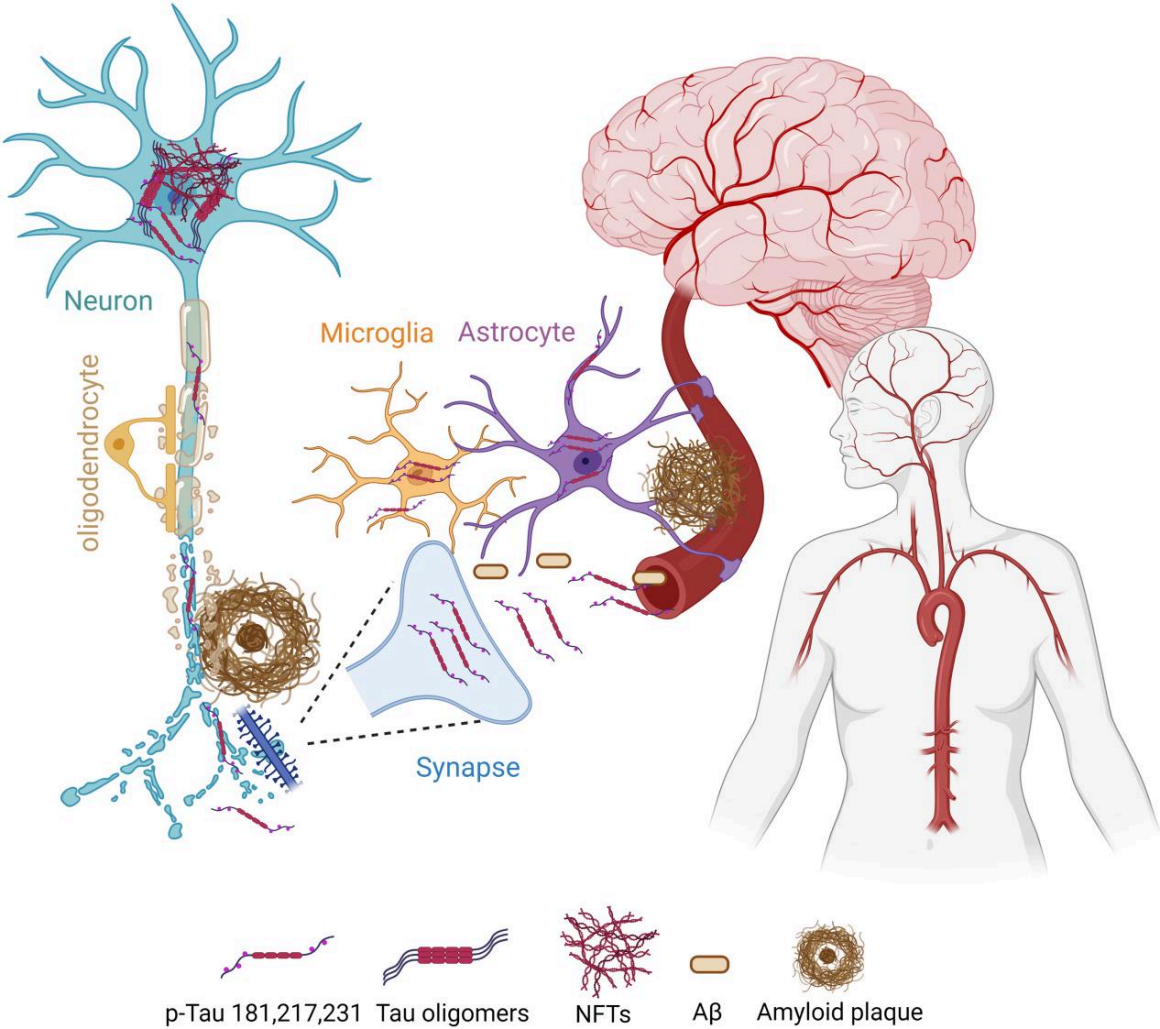
**Cellular localization of phosphorylated and aggregated tau in the brain during aging and Alzheimer’s disease.** The cellular distribution of aggregated and phosphorylated tau species (p-tau: Thr 181, 217 and 231) in the brain during aging and Alzheimer’s disease is shown. Synaptic and neuritic p-tau and Aβ released to the extracellular space are captured by glial cells (microglia and astrocytes) and they could be the source of tau and Aβ species found in cerebral and peripheral biofluids (CSF and blood).
